# Postpartum Guillain-Barré Syndrome Presenting as Acute Motor Axonal Neuropathy in a Young Female: A Report of a Rare Case

**DOI:** 10.7759/cureus.70787

**Published:** 2024-10-03

**Authors:** Muhammad Maqbool, Kuchi Deekshitha, Doddapaneni D Chandana, Zaghum Abbas, Ananya Talukdar, Asma Mehdi

**Affiliations:** 1 Internal Medicine, Shaheed Mohtarma Benazir Bhutto Medical College Lyari, Karachi, PAK; 2 Internal Medicine, Sri Venkateswara Medical College, Tirupati, IND; 3 Nephrology, Bahawal Victoria Hospital, Bahawalpur, PAK; 4 Neurology, Bharati Vidyapeeth Medical College, Pune, IND; 5 Surgery, Rawalpindi Medical University, Rawalpindi, PAK

**Keywords:** acute motor axonal neuropathy, aman, corticosteroid therapy in gbs, immune-mediated polyneuropathy, motor weakness, nerve conduction studies, neurological complications in pregnancy, plasmapheresis in gbs, postpartum neuropathy

## Abstract

This case report presents a rare instance of acute motor axonal neuropathy (AMAN), a subtype of Guillain-Barré syndrome (GBS), in a 25-year-old postpartum female. The patient experienced a seven-day history of progressive, ascending motor weakness in all four limbs, beginning shortly before delivery and rapidly worsening postpartum. Notably, she exhibited no sensory deficits, a hallmark feature of AMAN, which was confirmed by nerve conduction studies revealing significant motor axonal involvement with preserved sensory function. The diagnostic process was complicated by the overlap of AMAN symptoms with other neurological conditions and the unusual postpartum context. Treatment with high-dose corticosteroids and plasmapheresis resulted in significant clinical improvement, underscoring the effectiveness of these interventions in managing AMAN.

This case highlights the importance of considering AMAN in postpartum patients presenting with acute motor deficits, even in the absence of typical GBS sensory symptoms. It also emphasizes the need for early diagnosis and a multidisciplinary approach to optimize patient outcomes. By contributing to the limited literature on AMAN in postpartum women, this report aims to enhance clinical awareness, improve diagnostic accuracy, and inform treatment strategies for similar cases in the future.

## Introduction

Guillain-Barré syndrome (GBS) is a rare but serious acute immune-mediated polyneuropathy that primarily affects the peripheral nervous system, causing progressive weakness and, in some cases, life-threatening complications. GBS includes several subtypes, with acute motor axonal neuropathy (AMAN) representing a distinct variant. AMAN is characterized by the selective involvement of motor axons while sparing sensory nerves, clinically presenting as pure motor weakness without sensory loss [1]. This variant accounts for 5-10% of GBS cases in North America and Europe, but its prevalence is significantly higher in regions such as East Asia and South America, where it constitutes 30-65% of cases [2]. The pathophysiology of AMAN is believed to involve immune-mediated damage to the axolemma of motor nerves, often triggered by a preceding infection, particularly with *Campylobacter jejuni* [3].

Clinically, AMAN presents with a rapid onset of motor weakness that is often symmetrical and predominantly affects the lower extremities before ascending to involve the upper limbs. Unlike other variants of GBS, sensory symptoms are minimal or absent, and reflexes may be preserved in some cases [3]. Epidemiologically, AMAN is more frequently observed in children and young adults, with a slightly higher male predilection (male-to-female ratio of approximately 1.5:1) [1]. The clinical course of AMAN can be more aggressive, with faster progression and a slower recovery compared to other GBS variants, although long-term outcomes can be favorable with appropriate and timely intervention.

The objective of this case report is to describe a rare presentation of AMAN in a postpartum young female, an unusual demographic for this condition. The report aims to detail the distinctive clinical features, the diagnostic challenges encountered, and the management strategies employed in this unique case. By contributing to the limited literature on AMAN in postpartum patients, this case report seeks to enhance clinical awareness and improve diagnostic accuracy and treatment strategies for similar presentations in the future.

## Case presentation

A 25-year-old female presented to the hospital with a seven-day history of progressive weakness affecting all four limbs. The weakness began insidiously, initially manifesting in the lower limbs with difficulty in foot movement, which then advanced to a complete inability to move her feet. Over the following days, the weakness ascended to involve the upper limbs, resulting in an inability to grip or move her fingers. The patient denied any associated symptoms such as urinary or fecal incontinence, headache, or fever.

Further history revealed that the patient had experienced a febrile illness with a cough approximately 14 days prior to the onset of her neurological symptoms. Notably, she had delivered a healthy baby boy via spontaneous vaginal delivery (SVD) five days before presentation, with the weakness beginning two days before delivery. There was no personal or family history of neurological disorders or similar symptoms.

On neurological examination, the patient was fully alert and oriented with a Glasgow Coma Scale (GCS) score of E4V5M6 (15/15). The GCS is a neurological scale used to assess a patient's level of consciousness by evaluating eye opening (E), verbal response (V), and motor response (M). In this case, the patient had spontaneous eye opening (E4), was oriented and able to respond verbally (V5), and exhibited a normal motor response to commands (M6), resulting in the highest possible score of 15/15, which indicates full alertness. A summary is given in Table 1.

**Table 1:** A summary of the patient's neurological status based on the Glasgow Coma Scale.

Category	Score	Description
Eye opening (E)	4	Spontaneous (E4)
Verbal response (V)	5	Oriented (V5)
Motor response (M)	6	Obeys commands (M6)

Cranial nerve examination was normal, and there were no signs of cranial nerve deficits. The plantar reflexes were bilaterally static, and muscle tone was normal in both the upper and lower extremities. However, deep tendon reflexes were absent in the lower limbs and markedly diminished in the upper limbs. Muscle power was graded at 0/5 in both upper and lower limbs, indicating profound weakness. Sensory examination revealed no deficits; sensation was intact in all dermatomes, and the pupils were equal, dilated, and reactive to light.

Given the clinical presentation of ascending motor weakness with preserved sensory function and the absence of reflexes, there was a high suspicion for GBS, specifically the AMAN variant. To confirm the diagnosis and assess the extent of the condition, a series of diagnostic investigations were undertaken.

Initial laboratory investigations were conducted to evaluate the patient's overall health status and to rule out any underlying conditions that could contribute to her neurological symptoms. The relevant findings are summarized in Table 2 below, highlighting significant abnormalities, including mild microcytic anemia and elevated liver enzymes, which may be indicative of recent physiological stress, such as postpartum status or a preceding infection.

**Table 2:** Hematology and biochemistry results of the patient.

Investigation	Result	Normal reference range
Hemoglobin	11.3 g/dL	13-18 g/dL
Hematocrit	34.4%	35-46%
Mean corpuscular volume	69 fl	77-95 fl
Mean corpuscular hemoglobin	22.7 pg	26-32 pg
Alanine transaminase	74.4 U/L	Up to 40 U/L
Aspartate transaminase	54.5 U/L	Up to 40 U/L
Urine color	Turbid yellow	Clear to pale yellow
Urine protein	Trace	<150 mg/dL
Urine pH	8	4.6-8

The nerve conduction studies demonstrated significant abnormalities consistent with the AMAN variant of GBS. The motor nerve conduction studies revealed markedly prolonged latencies and reduced amplitudes in multiple nerves, particularly in the tibial and peroneal nerves, indicating severe motor axonal involvement. Sensory nerve conduction studies showed relatively preserved sensory function with normal latencies and amplitudes, further supporting the motor-selective pathology of AMAN. Additionally, the absence of F-waves across all tested nerves suggests a proximal conduction block or severe axonal degeneration, confirming the diagnosis of AMAN. These findings align with the clinical presentation of rapidly progressive, purely motor weakness without sensory loss. The detailed nerve conduction studies are provided in Table 3, Table 4, and Table 5.

**Table 3:** Motor nerve conduction study findings.

Nerve	Site	Latency (ms)	Amplitude (mV)	Conduction velocity (m/s)	Normal reference range
Median (R)	Wrist	4.2	0.8	-	Latency: <3.6 ms, amplitude: >4.0 mV
	Elbow	8.6	0.7	48	Latency: <6.5 ms, conduction: >50 m/s
Ulnar (R)	Wrist	3.8	0.6	-	Latency: <3.1 ms, amplitude: >6.0 mV
	Below the elbow	7.9	0.5	52	Latency: <6.0 ms, conduction: >55 m/s
Tibial (R)	Ankle	5.6	0.3	-	Latency: <4.4 ms, amplitude: >3.5 mV
	Popliteal fossa	15.2	0.2	38	Latency: <12.5 ms, conduction: >40 m/s
Peroneal (R)	Ankle	5.2	0.2	-	Latency: <5.5 ms, amplitude: >3.0 mV
	Fibular head	13.8	0.1	40	Latency: <10.0 ms, conduction: >45 m/s

**Table 4:** Sensory nerve conduction study findings.

Nerve	Site	Latency (ms)	Amplitude (µV)	Conduction velocity (m/s)	Normal reference range
Median (R)	Wrist-digit II	3.2	18	56	Latency: <3.5 ms, amplitude: >20 µV
Ulnar (R)	Wrist-digit V	3	15	54	Latency: <3.1 ms, amplitude: >15 µV
Sural (R)	Calf-lateral malleolus	3.8	12	48	Latency: <4.0 ms, amplitude: >6 µV

**Table 5:** F-wave study results.

Nerve	F-wave latency (ms)	F-wave persistence (%)	Normal reference range
Median (R)	Absent	0%	Latency: <32 ms, persistence: >70%
Ulnar (R)	Absent	0%	Latency: <31 ms, persistence: >70%
Tibial (R)	Absent	0%	Latency: <50 ms, persistence: >70%

Upon confirming the diagnosis of AMAN, the patient was promptly initiated on a high-dose corticosteroid regimen with intravenous methylprednisolone, administered at 250 mg every six hours (four times a day (QID)). This treatment was aimed at mitigating inflammation and preventing further deterioration of motor function. Given the risk of respiratory compromise, chest expansion was monitored rigorously every two hours to manage any potential respiratory distress.

In conjunction with corticosteroid therapy, the patient was prepared for plasmapheresis, a critical intervention for accelerating recovery in AMAN. To date, three sessions of plasmapheresis have been completed, resulting in notable clinical improvement. Muscle strength in both upper and lower limbs has increased to 3/5. The patient is scheduled for two additional plasmapheresis sessions, and a comprehensive physiotherapy program has been initiated to enhance functional recovery and prevent complications related to immobilization.

The importance of adherence to the treatment regimen, including attending all scheduled plasmapheresis sessions and participating in physiotherapy, was emphasized to maximize her chances of a full recovery. Additionally, she was advised to monitor and report any changes in her condition, particularly respiratory difficulties, to ensure timely intervention. Regular follow-ups will be crucial to assess her progress, adjust treatments as needed, and provide ongoing support and guidance throughout her recovery process.

## Discussion

This case report describes a rare presentation of AMAN, a variant of GBS, in a postpartum young female. The patient exhibited progressive motor weakness without sensory loss, beginning in the lower limbs and advancing to the upper limbs shortly after childbirth. Diagnostic investigations, including nerve conduction studies, confirmed the diagnosis of AMAN. The patient was managed with high-dose corticosteroids and plasmapheresis, leading to significant clinical improvement. This case is significant because it highlights the uncommon occurrence of AMAN in the postpartum period, underlining the need for heightened clinical awareness and tailored management strategies for similar presentations.

The presentation of AMAN in this postpartum patient is an unusual finding, as existing literature predominantly reports AMAN cases in children and young adults, often following a preceding infection. Compared to other reported cases, the absence of sensory symptoms and preserved reflexes in this patient aligns with typical AMAN characteristics, but the postpartum onset is exceptionally rare. Epidemiologically, AMAN is more common in East Asia and South America, with fewer cases in postpartum females in Western literature. This case expands the geographic and demographic understanding of AMAN, emphasizing the need to consider this diagnosis in postpartum patients presenting with acute motor deficits, even in regions where it is less common.

Diagnosing AMAN in a postpartum patient presented unique challenges due to the overlap of its symptoms with other GBS subtypes and neurological deficits commonly associated with postpartum complications [4]. Differentiation from other GBS variants was particularly challenging given the lack of sensory symptoms and preserved reflexes in some cases. The use of nerve conduction studies and F-wave studies was crucial in identifying the distinct motor axonal involvement characteristic of AMAN, with findings of reduced motor amplitudes and absent F-waves providing definitive evidence to confirm the diagnosis and differentiate it from other conditions with similar presentations [5].

The onset of AMAN in the postpartum period may be explained by several pathophysiological mechanisms. Immune system alterations during pregnancy and the postpartum period, such as immune tolerance and subsequent rebound immune activation, could predispose individuals to autoimmune conditions like AMAN. Furthermore, the patient's history of a febrile illness preceding neurological symptoms suggests a possible link to infections, such as *Campylobacter jejuni*, known to trigger autoimmune responses that attack peripheral nerves. These factors collectively may contribute to the development of AMAN in a previously healthy postpartum female.

The treatment strategy combining high-dose corticosteroids and plasmapheresis was effective in managing this case, aligning with the standard practices for treating severe GBS variants but tailored to the patient's postpartum status. The corticosteroids helped reduce inflammation, while plasmapheresis was crucial in rapidly removing circulating antibodies, resulting in significant clinical improvement. Compared to typical outcomes for AMAN patients, particularly in non-postpartum settings, this patient demonstrated a relatively swift recovery, suggesting that early intervention and aggressive management can lead to favorable outcomes even in atypical presentations [6].

This case underscores the importance of recognizing atypical presentations of AMAN, particularly in rare demographics such as postpartum women, which can lead to earlier diagnosis and timely intervention, ultimately improving patient outcomes. Healthcare providers should maintain a high index of suspicion for AMAN when encountering postpartum patients with acute motor deficits, even in the absence of typical sensory symptoms or reflex changes. Practical recommendations for managing such cases include prompt referral to a neurologist for advanced diagnostic testing, close monitoring of respiratory function, and the initiation of appropriate therapies such as corticosteroids and plasmapheresis. A multidisciplinary approach, involving neurologists, obstetricians, and rehabilitation specialists, is crucial to provide comprehensive care and support, enhancing recovery and minimizing long-term disability in affected patients.

## Conclusions

This case report highlights a rare presentation of AMAN in a postpartum young female, underscoring the need for heightened clinical awareness among healthcare providers. The unique demographic context, coupled with the absence of sensory symptoms and the rapid progression of motor weakness, posed significant diagnostic challenges. However, early recognition and prompt intervention using high-dose corticosteroids and plasmapheresis resulted in a favorable clinical outcome, emphasizing the importance of timely diagnosis and tailored management in improving patient recovery. This case reinforces the value of considering atypical presentations of AMAN in postpartum patients, advocating for a multidisciplinary approach to optimize care and patient outcomes.
